# The seismicity of Campi Flegrei in the contest of an evolving long term unrest

**DOI:** 10.1038/s41598-022-06928-8

**Published:** 2022-02-21

**Authors:** Anna Tramelli, Flora Giudicepietro, Patrizia Ricciolino, Giovanni Chiodini

**Affiliations:** 1 Istituto Nazionale di Geofisica e Vulcanologia, Osservatorio Vesuviano, Via Diocleziano 328, 80124 Naples, Italy; 2 Istituto Nazionale di Geofisica e Vulcanologia, Sezione di Bologna, Via Donato Creti, 12, 40128 Bologna, Italy

**Keywords:** Natural hazards, Solid Earth sciences

## Abstract

One of the most effective approaches to identifying possible precursors of eruptions is the analysis of seismicity patterns recorded at volcanoes. Accurate locations of the seismicity and the estimate of source mechanisms can resolve fault systems and track fluid migrations through volcanoes. We analysed the six main swarms recorded at Campi Flegrei since 2000, using them as a proxy of the processes involved in the long-term-unrest of this densely populated caldera. We re-located the earthquakes comprised in these swarms and estimated the focal mechanisms, which appear in agreement with the fault systems of the caldera and with tomographic images. The focal mechanisms are in agreement with the tensional stress induced by the caldera uplift. Most of the swarms and remaining seismicity delineate a highly fractured volume extending vertically below the Solfatara/Pisciarelli vents, where gases find preferential paths to the surface triggering earthquakes. The main swarms are located below this volume where the presence of a rigid caprock is still debated. We interpreted the current unrest in term of a gradual increment in the activity of a wide hydrothermal system whose most evident manifestation is the enlargement of the fumarolic-field of Pisciarelli and the increment of the earthquakes occurrence rate.

## Introduction

The Campi Flegrei caldera, southern Italy, is one of the most populated active volcanoes on the Earth. Its ground suffers repeated vertical uplifts and subsidence phases; since 1950, four episodes of uplift (bradyseism crises) that raised the coastal town of Pozzuoli by up to 4 m^1,2^ are reported. The uplift episodes are believed to be governed by overpressure of the shallow hydrothermal system^3–5^, magma movement through the crust^6^ or by both the phenomena^7,8^. The seismicity which characterizes the area is shallow, low in magnitude (up to Md = 4.0 on March 14, 1984) and mainly located below the area of Solfatara/Pisciarelli^5,9–12^.

Few LP events were identified during the bradiseismic crises of 1982–84^13^. In 2000, when the last phase of subsidence stopped, a swarm of hybrid events was recorded in the area South of Solfatara at a depth of almost 3 km. These events showed a dominant frequency around 1–3 Hz and a clear S-wave onset, placing them in the hybrid category^13^. In October 2006, a shallow LP activity accompanied by a VT swarm^13,14^ lasted for 1 week.

During the last bradiseismic crisis, in 1982–84, more than 16,000 earthquakes accompanied an uplift of almost 1.8 m. In this period, seismic swarms have been identified which correlate in space and time with possible fluid injection from a deep hot source^15^. The swarms’ locations, compared with the attenuation topography images, were interpreted in terms of fluid migration. The fluids moved from a volume located 3–4 km below Pozzuoli toward a shallower aseismic volume below Solfatara, identified as the ground deformation source, reaching the area of the last eruption (Mt. Nuovo, 1538) at the end of the crisis^15^. During this crisis an offshore WNW-ESE seismogenic structure southwest of Pozzuoli was identifies^13^. After 1984, the caldera had subsided continuously, except for occasional minor uplift episodes, until 2000 when the trend slightly reversed^1,13^. At that time two swarms (the hybrid events mentioned above and one comprising exclusively VT earthquakes) were recorded after a prolonged aseismic period. The seismicity recorded since 2000 was located mostly onshore and concentrates at Solfatara/Pisciarelli^9^. Very few events occured offshore and the WNW-ESE structure that was identified in the previous crisis (1982–84) was practically aseismic.

After the eruption of the Neapolitan Yellow Tuff (about 15 ky B.P.), Campi Flegrei caldera was affected by variable vertical movements of disjointed blocks with fault and fracture systems mainly trending NE–SW and NW–SE^16^. Many vents active over the past 15,000 years have been identified within the caldera^17^. Their areal distribution with time is a good tracer of the active structures which have favoured magma movement towards the surface^7,18^. In the recent eruptive history of Campi Flegrei, stress changes, due to topographic, bathymetric and density variations between the caldera centre and periphery and to the inflating central source, favoured the magma movement towards different faults reaching the surface in different areas within the caldera^19^.

The ground deformations recorded during the last bradiseismic crises have been associated to both magmatic or hydrothermal sources and their location, depth, shape and density have been constrained thanks to gravimetric (^20^ and references therein), seismological (e.g.^6^), pore pressure^21^, geochemical or petrological measurements^22^. A deep magmatic source is usually invoked as the source for magma movements towards the surface (e.g.^23^) or to justify the movement of shallow hydrothermal fluids. Fluid movements were evidenced during the 1982–1984 crisis (e.g.^15^) and also during the current unrest (e.g.^11,24,25^) and their intrusion were linked to the characteristic of the caprock present at 2–3 km depth^26^.

We investigated the swarm seismicity recorded at Campi Flegrei in the last 20 years to obtain information on faults, stress regimes and fluid movements that are mainly involved during the current uplift phase and to draw considerations on the possible preferential routes for fluids and magma movements towards the surface.

## Results

**Figure 1:**
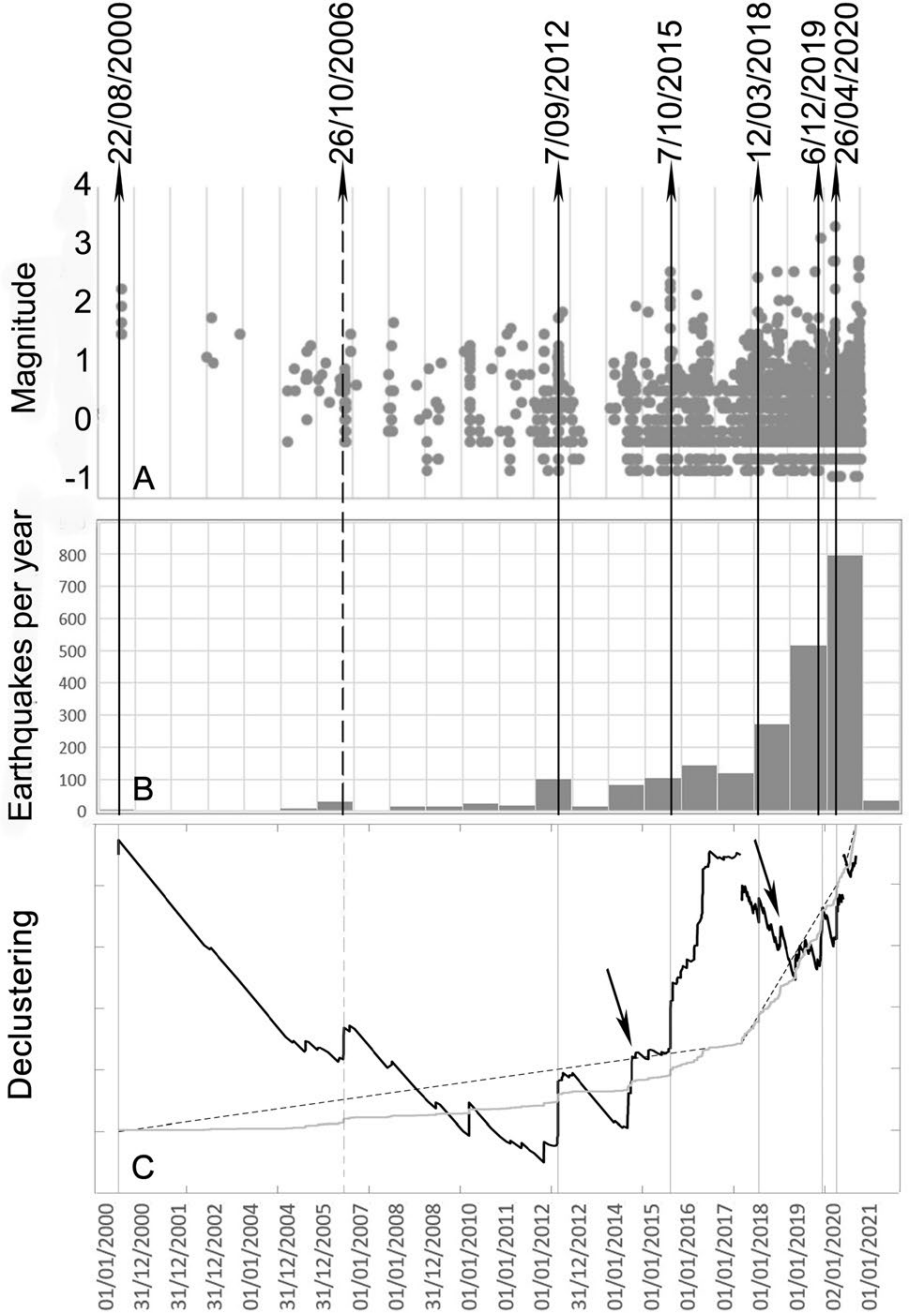
Earthquakes magnitudes (**A**) and annual occurrence (**B**) recorded in the Campi Flegrei caldera from January 2000 to March 2021. Vertical rows indicate the 6 main swarms analyzed in this article and the main LP swarm (dashed) recorded by the seismic network of Osservatorio Vesuviano, INGV. (**C**) The declustering functions in normalized units (black bolds lines) compared with the cumulative number (grey line) and the expected number of earthquakes based on the estimated rate (dashed lines).

Figure 1B shows the monthly seismicity and the earthquake magnitudes recorded at Campi Flegrei from 2000 to March 2021 by the seismic network of Osservatorio Vesuviano, INGV. As evidenced by Tramelli et al.^4^ the occurrence rate of earthquakes increased with time since 2000 and the hypocentral depth is decreasing. We analysed 6 main swarms (black vertical lines in Fig. 1, summary in Table 1) and described their locations and focal mechanisms in the context of the whole seismicity, the vertical movement of the ground, the known faults and fractures and the subsoil structure.

**Table 1:** Summary of the analyzed swarms: swarm occurrence day, number of detected earthquakes, maximum and minimum magnitude and elevation rate (from monthly or weekly bulletins of INGV).

Day	N°	M_max_	M_min_	Elevation rate
22/08/2000	90	2.1	− 0.5	~ 0.7 cm/month
7/09/2012	180	1.7	− 1.1	1–1.6 cm/month
7/10/2015	33	2.5	− 1.1	1.5 cm/month
12/03/2018	48	2.4	− 0.8	0.7 cm/month
6/12/2019	34	3.1	− 1.1	1 cm/month
26/04/2020	81	3.3	− 1.1	0.6–0.7 cm/month

### August 22, 2000

**Figure 2:**
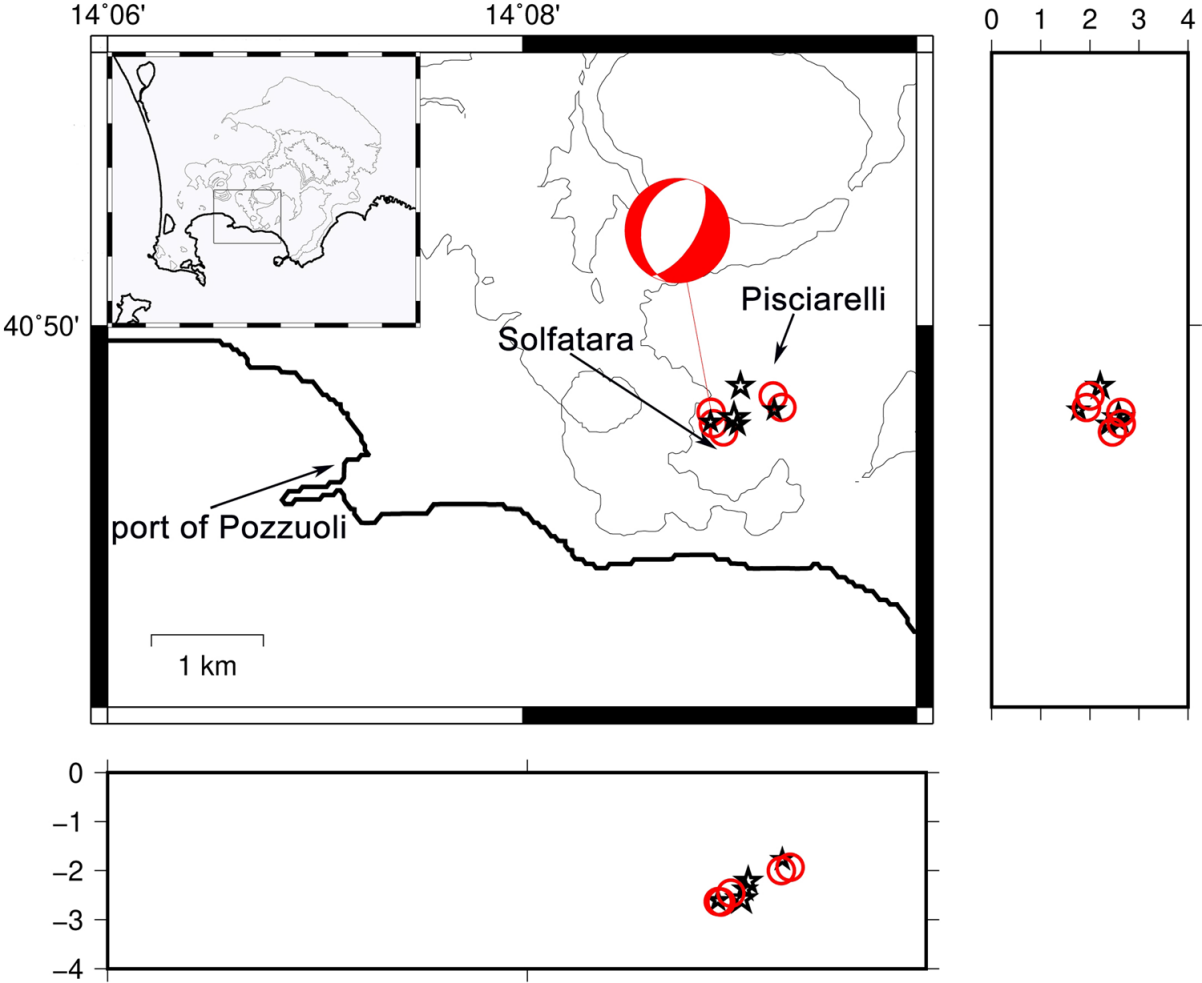
Location of the earthquakes of the swarm of 22/08/2000 obtained using the NLLoc program with a 3D velocity model^27^ (grey circles) and the HypoDD program in a 1D velocity model (black stars). Beach ball shows the focal mechanism of the Md = 2.1 earthquake. The figure was obtained using the free software GMT 5.3.1 (https://www.generic-mapping-tools.org/).

A swarm of VT events was recorded on August 22, 2000, a month after a swarm of hybrid events that lasted a week (July 2–7)^14^. 90 events with magnitudes between -0.5 and 2.1 were recorded within 7 h. Due to the number and position of the seismic stations at that time only 6 of those events were located. The hypocenters resulted below the Solfatara crater at a depth of about 2 km. To understand the sensitivity of the seismic network at that time, a detailed analysis is presented in Tramelli et al.^4^. The events occurred during a period of uplift started a couple of months before. The relocation of the earthquakes with both methods, NLLoc and HypoDD, shows a sub-vertical structure, which slightly tends to East with decreasing depth, extending between 2.6 and 1.9 km below Sofatara/Pisciarelli (Fig. 2). Since these events are very old and being complicated to find all the traces, we considered just the well located ones: horizontal and vertical location errors ≤ 100 m. The focal mechanism of the highest-magnitude earthquake is associable to a normal fault trending N-NE.

### September 7, 2012

The quasi-quiescence of the volcano between 2000 and 2012 was interrupted in October 2006 by a seismic swarm of LP events^14^, which we do not take into consideration here. After this LP swarm, on September 7, 2012 more than 180 low-magnitude earthquakes occurred within a time window of almost 3 h; two of them had the maximum magnitude of Md = 1.7. This was the largest swarm in terms of energy released and number of recorded earthquakes since the end of the bradiseismic crisis of 1982–84. The swarm occurred at the end of a period of almost 2 months of increment in the ground uplift rate, which passed from 0.4–0.5 cm/year to 1–1.6 cm/month in the central part of the caldera. Immediately after the swarm the ground uplift slowed down.

**Figure 3:**
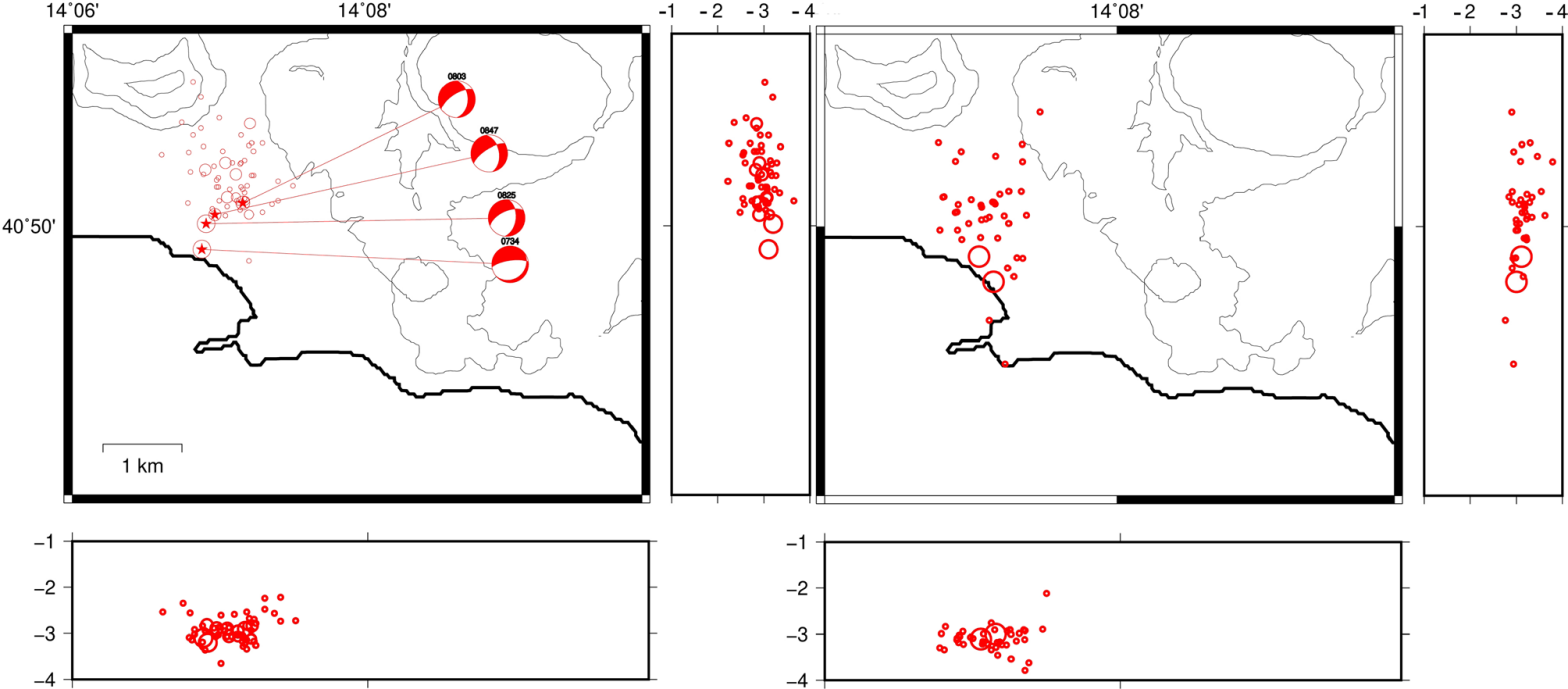
Location of the earthquakes of the swarm of 7/9/2012 using NLLoc (sx) and HypoDD (dx). Circle sizes are proportional to the earthquake magnitude. The beach balls of focal mechanisms are obtained using the program FPFit. The images were obtained using the free software GMT 5.3.1 (https://www.generic-mapping-tools.org/).

This swarm was located North to the area of the maximum uplift (port of Pozzuoli), differently from the seismicity recorded after 1984^13^, which is located close to the Solfatara/Pisciarelli area (NE to the maximum uplift area) (Fig. 3).

We relocated 64 earthquakes of the swarm in a very narrow volume of 2 × 2 km^2^ extending between 2.2 and 4.2 km of depth (Fig. 3). The mean location error was 150 m for the horizontal and less than 100 m for the vertical. The differences between the locations obtained with NLLoc and with HypoDD are not appreciable. The focal mechanisms are all normal and generally trending NE-SW.

### October 7, 2015

33 low magnitude earthquakes were recoded within a time window of almost 2 h in the area East to the Sofatara border on October 7, 2015. The swarm started at 7:20 and the maximum magnitude earthquake, Md = 2.5, was recorded at 9:10 almost at the end of the swarm. Also in this case, the swarm was recorded at the end of a period of increment in the velocity uplift (~ 1.5 cm/month), which started almost 1 month before (at the beginning of September).

**Figure 4:**
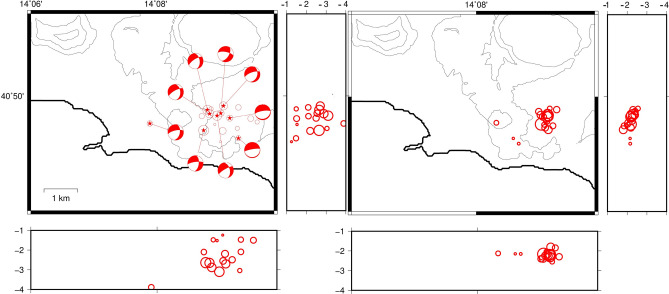
Location of the earthquakes of the swarm of 7/10/2015 using NLLoc (sx) and HypoDD (dx). Circle sizes are proportional to the earthquake magnitude. The images were obtained using the free software GMT 5.3.1 (https://www.generic-mapping-tools.org/).

The relocation of the earthquakes evidences an almost vertical structure extending below Solfatara/Pisciarelli at a depth of 1.2–3.9 km (Fig. 4); the mean horizontal and vertical location errors are < 350 m. The double difference location evidences a restricted hypocentral volume of ~ 1 km^3^. The focal mechanism solutions confirm the extensional field for the slipping fault.

### March 12, 2018

Between 13:33 and 15:35 of March 12, 2018, 42 seismic events were recorded by the seismic network of Osservatorio Vesuviano in the area of Solfatara-Pisciarelli. The maximum magnitude recorded was Md = 2.4, corresponding to the deepest earthquake of the swarm recorded at 14:09. Differently from the previous ones, this swarm occurred during a period of stable uplift, without any evidence of variation in the uplift rate which was almost 0.7 cm/month since middle 2017. Since 2014 the total uplift recorded at the GPS station located at the Pozzuoli harbor was almost 26 cm.

**Figure 5:**
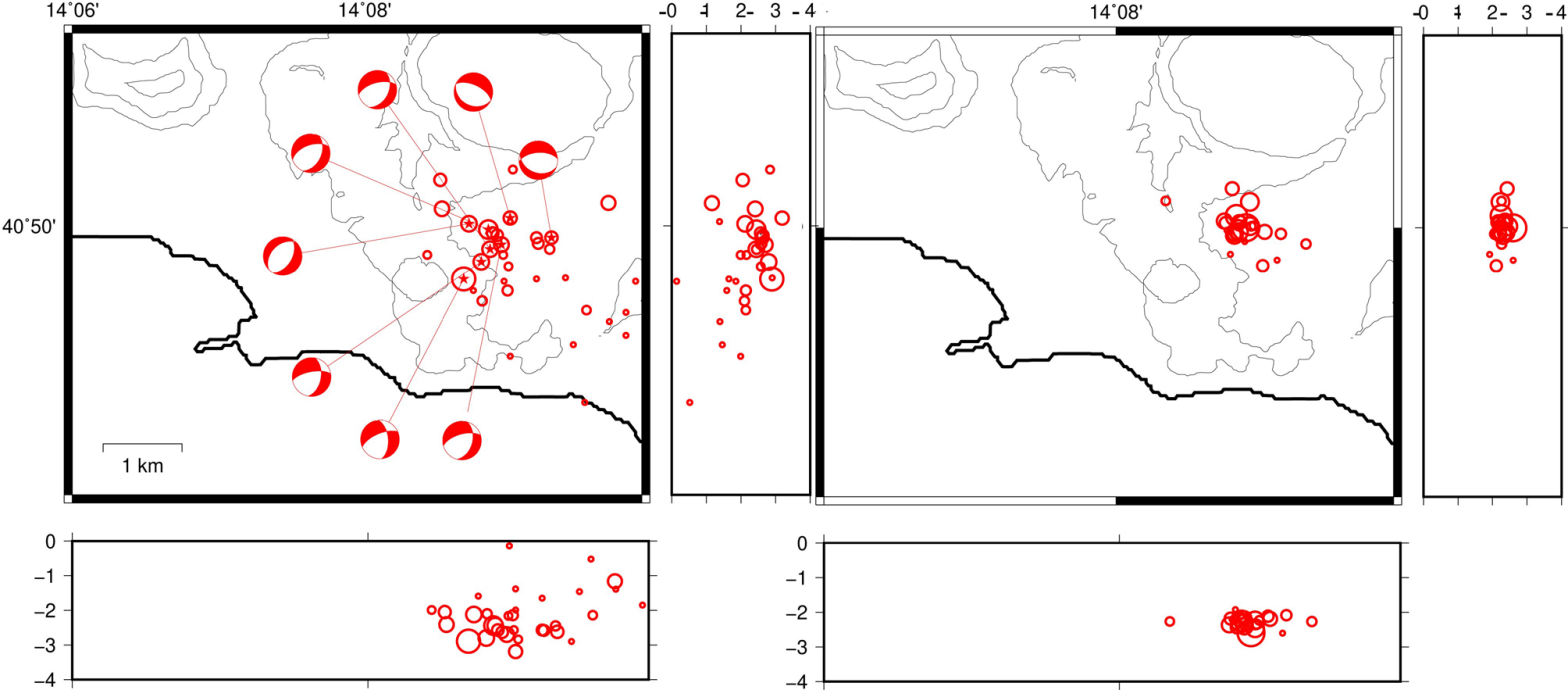
Location of the earthquakes of the swarm of 12/3/2018 obtained using NLLoc (sx) and HypoDD (dx). Circle sizes are proportional to the earthquake magnitude. The images were obtained using the free software GMT 5.3.1 (https://www.generic-mapping-tools.org/).

We relocated 35 earthquakes of the swarm. Using the program NLLoc the events result located in an area of almost 3 × 3 km^2^ between 0 and 3.2 km depth; with mean horizontal and vertical error of 100 m. The hypocentral volume is highly reduced using the double difference location resulting in a flattened shape of 0.5 × 0.5 km^2^ extending between 2 and 3 km of depth. The solutions of the focal mechanisms are all normal and trending primarily NE-SW (Fig. 5).

### December 5–6, 2019

34 earthquakes with maximum magnitude Md = 3.1 were recorded in the area of Solfatara/Pisciarelli between 24:51 of December 5 and 2:24 of December 6, 2019. The hypocenters were located between depths of 0.9 and 2.3 km. The uplift mean velocity showed an increment between the end of November and the beginning of December, passing from almost 7 mm/month to about 1 cm/month and moved back to the values of 7 mm/month after the swarm. At that time, the total uplift recorded in the center of the caldera since 2005 was 65 cm.

**Figure 6:**
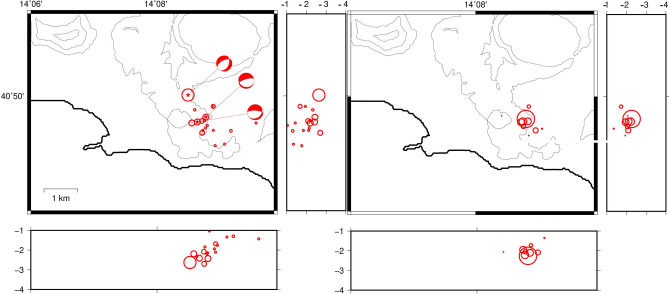
Location of the earthquakes of the swarm of 6/12/2019 obtained using NLLoc (sx) and HypoDD (dx). Circle sizes are proportional to the earthquake magnitude. The images were obtained using the free software GMT 5.3.1 (https://www.generic-mapping-tools.org/).

We relocated 18 earthquakes of the swarm and plot the results in Fig. 6. The locations obtained with NLLoc are spread in an area of ~ 2 × 3 km^2^ extending between 1 and 2.7 km depth with a mean horizontal and vertical error of 80 m. When applying the double-differences approach, the earthquakes of the swarm concentrate within a very small volume but is still visible a structure vertically elongated below Solfatare/Pisciarelli. The focal mechanisms are normal trending NE-SW.

### April 26, 2020

A seismic swarm was recorded between 1:26 and 5:23 of April 26, 2020. 81 earthquakes with low magnitude (max Md = 3.3) were identified. The most energetic event was recorded at 2:59 and was located below the Solfatara/Pisciarelli area at the depth of 2.6 km. This earthquake is the highest magnitude recorded from the end of the bradiseismic crises of 1982–84 until the end of 2021. The vertical uplift recorded at the center of the caldera had an increment between the end of November and the beginning of December 2019, when the previous swarm was recorded, but it come back to values of 6–7 mm/month before this swarm. No anomalies in the vertical displacement can be directly associated with this swarm. From 2005 to April 2020 a total uplift of almost 70 cm was recorded at the Pozzuoli harbor.

**Figure 7:**
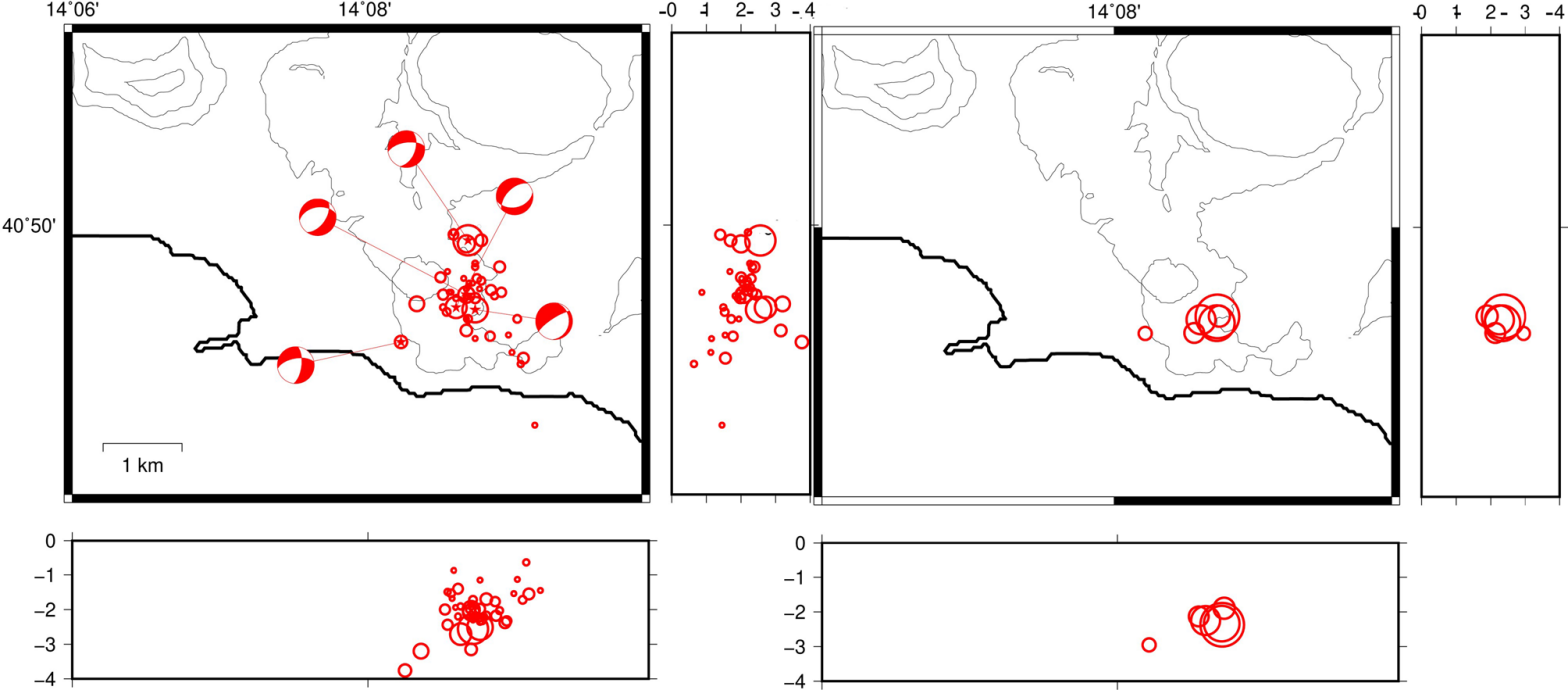
Location of the earthquakes of the swarm of 26/04/2020 obtained using (sx) and HypoDD (dx). Circle sizes are proportional to the earthquake magnitude. The images were obtained using the free software GMT 5.3.1 (https://www.generic-mapping-tools.org/).

We relocated 46 earthquakes of the swarm and the results are shown in Fig. 7. The locations and focal mechanisms are similar to those comprised in previous swarms. Also in this case the hypocentral volume estimated using the absolute locations obtained with NLLoc is wider than the one estimated using HypoDD. The mean horizontal and vertical location errors obtained with the Bayesian approach are ~ 100 m.

### The focal mechanisms

We analyzed the focal mechanisms of the earthquakes of the most significant swarms recorded since 2000 to evidence any variation in the local stress. We showed beach balls for earthquakes with sufficient azimuthal coverage and with adequate signal-to-noise ratios (usually Md ≥ 1). The rapid occurrence of earthquakes within a swarm sometimes obscures the first arrivals also for magnitudes higher than 1.5 and increases the picking errors.

The obtained focal mechanisms are all congruent (see supplementary Table 1): normal and oriented mostly NE-SW confirming the local extensional trend of the caldera, which is suffering a persistent uplift in its central part. The obtained results confirm what founded by La Rocca and Galluzzo^28^ for the events of the seismic catalogue of Campi Flegrei between 2005 and 2019. The earthquakes belonging to the swarms are all located below the area of Solfatara/Pisciarelli at 2–4 km, except for the 2012 swarm. This swarm was located North of the area of maximum uplift at a depth of almost 3 km. Furthermore, the location of the earthquakes belonging to the 2012 swarm evidence a NS direction and a squashed hypocentral volume. After this swarm, this volume was not involved anymore in the seismicity of the caldera.

**Figure 8:**
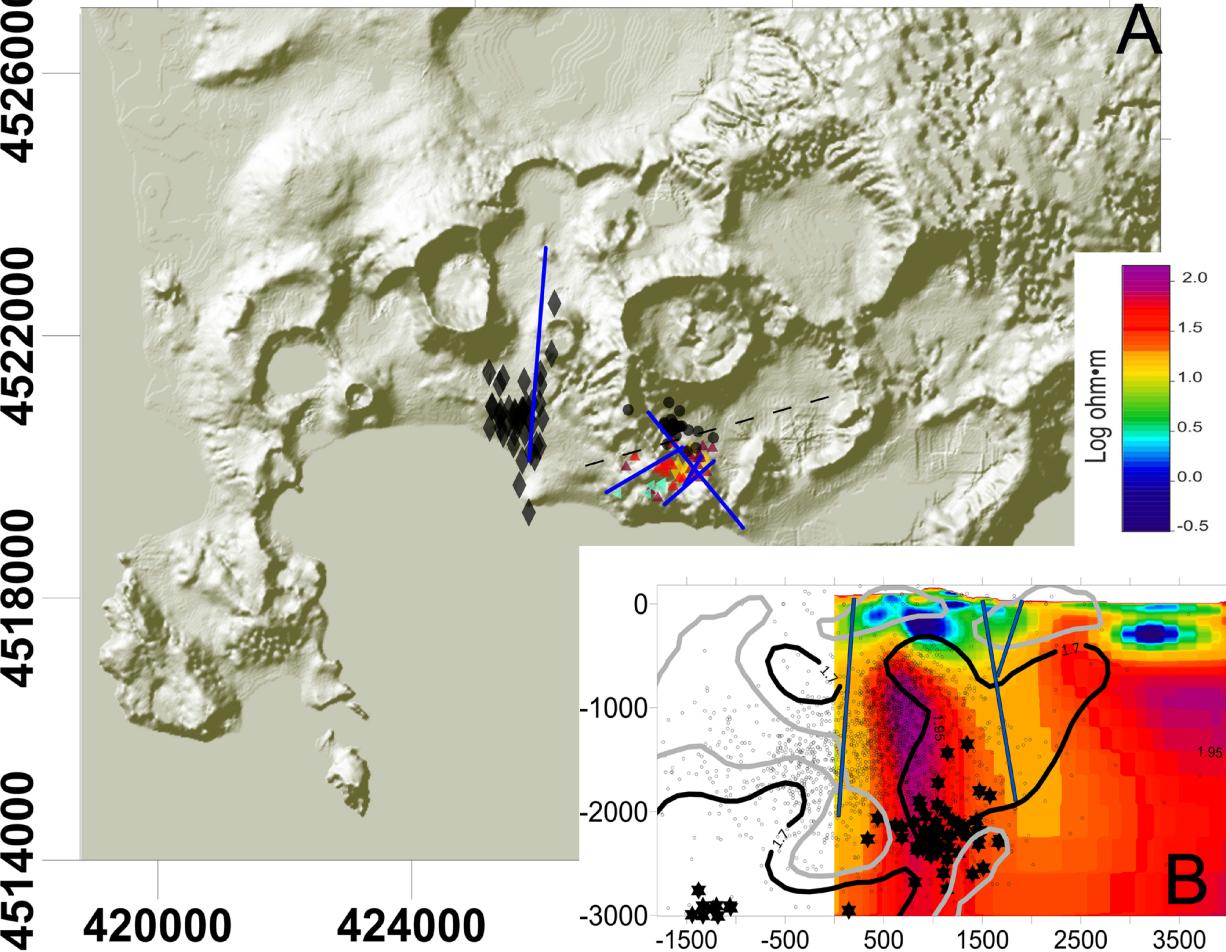
Slide A: Plot of the Campi Flegrei map with the indication of faults (blue lines) that overlap with the relocated swarms. The faults are taken by Vilardo et al.^29^. The swarms are: 2000-yellow diamonds; 2012-black diamonds; 2015—Bordeaux filled triangles; 2018—black filled circles; 2019—red filled triangles; 2020—left oriented light blue triangles. Black dashed line indicate the vertical projection of the resistivity tomography reported in slide B. Slide A was obtained using the software Surfer 22 (https://www.goldensoftware.com/products/surfer). Slide B: resistivity model modified after Siniscalchi et al.^30^ adding the relocated events (black dots), swarms (black stars) and low (< 1.7) and high (> 1.9) Vp/Vs areas (black contours and grey contours, respectively). With blue line we reported the faults deduced by Siniscalchi et al.^30^ thanks to the resistivity image.

Changes in the differential stress between the caldera centre and periphery, due to topographic, bathymetric and density variations^31^, make a correlation between the stress induced by the inflating of the central part of the caldera and the orientation of the focal mechanisms largely ineffective. Pepe et al.^32^ inferred that stress, seismicity and secondary deformation are likely modulated by shallow structures, located at a depth of ~ 1 km, under the eastern sector of the caldera. In the same paper, the authors also evidenced that shallow structures are able to channel fluids in the Solfatara/Pisciarelli area. Regional stress^33^ and pre-existing tectonic structures play, indeed, an important role on the modulation of the stress distribution^34^. More efficient is the comparison of the direction of the mechanisms with the known faults system distributed within the caldera. We plotted the relocated swarms on a simplified map of the faults and fractures presented by Vilardo et al.^29^ (Fig. 8A) to evidence any alignment. All the earthquakes of the swarms occurred along the faults that cut the Solfatara/Pisciarelli area with SW-NE orientation and on the orthogonal ones. The focal mechanisms are congruent with the SW-NE orientation. Again, the only exception is the 2012 swarm. In this case the earthquake locations follow the N-S fault that cuts the caldera from the South of San Vito area to the port of Pozzuoli (Fig. 8). The focal mechanisms are oriented NE-SW and the hypocenters are the deepest recorded at Campi Flegrei since 2000.

### The swarms in the context of the whole seismicity

**Figure 9:**
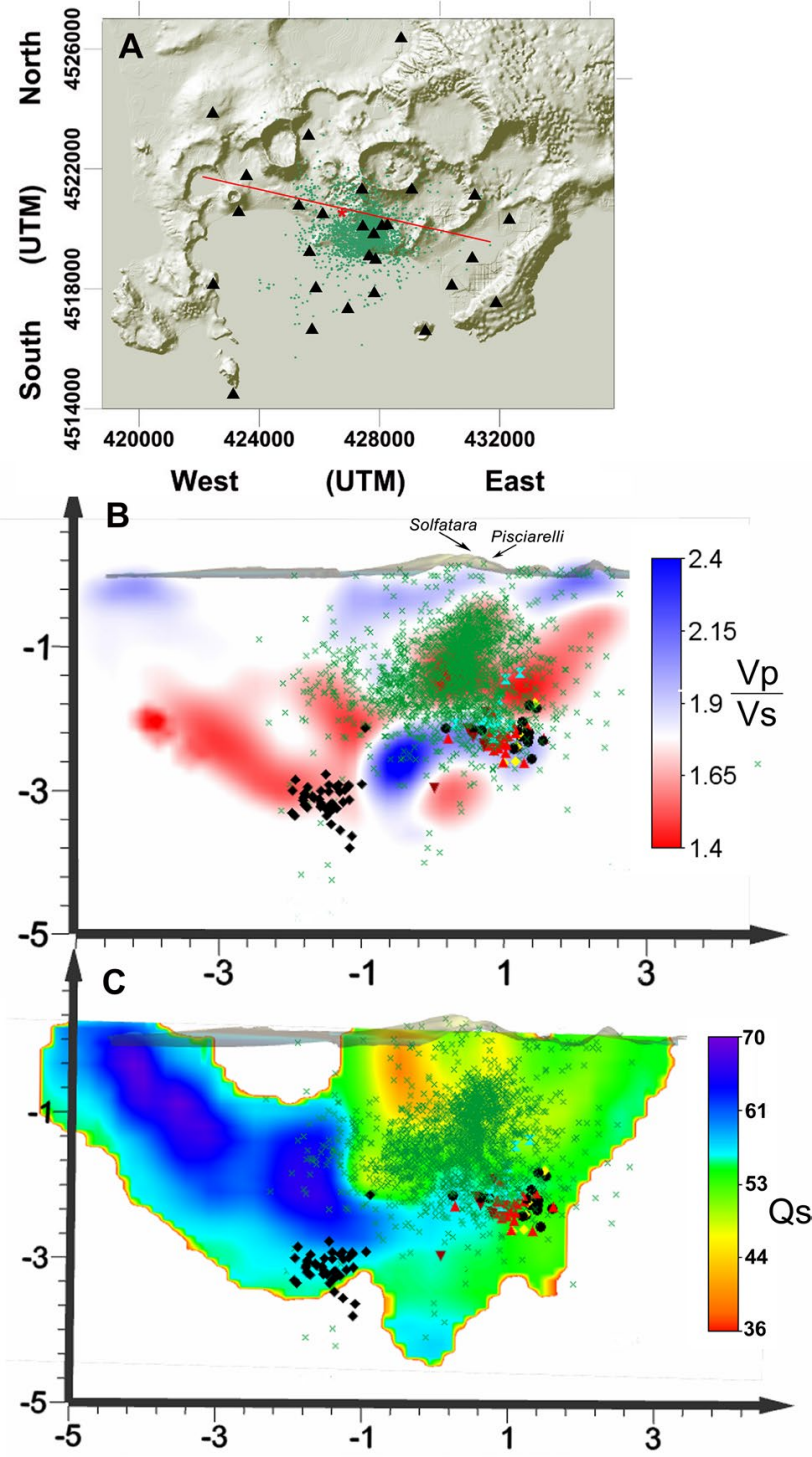
(**A**) Map of the Campi Flegrei caldera with epicenters of earthquakes recorded from 2000 to the end of 2020. Black triangles are the seismic stations of the current seismic network. Red line indicates the surficial projections of the slides B and C. (**B**) Vertical projection of Vp/Vs tomography with the earthquakes of the swarms of 2012, 2015, 2018, 2019 and 2020. To indicate the different swarms, we used different black symbols: 2000—yellow diamonds; 2012—black diamonds; 2015—bordeaux reversed triangles; 2018—black filled circles; 2019—red filled triangles; 2020—hourglasses. Green crosses indicate the relocated earthquakes of the seismic catalogue between 2000 and 2020. (**C**) Vertical projection of the attenuation tomography with the hypocenters of the five analyzed swarms and the earthquakes of the relocated seismic catalogue (green crosses). Slide A was obtained using the software Surfer 22 (https://www.goldensoftware.com/products/surfer). Slides B and C were built with the software Voxler 3 (http://www.goldensoftware.com/products/voxler).

Most of the seismicity recorded since 2000 is located below the area of Solfatara/Pisciarelli, NE to the area of maximum uplift, with maximum hypocentral density at 0.5–1 km (Fig. 9B)^35^.

The relocation of the whole seismicity and of the main swarms with a 3D velocity model^27^ and a Bayesian approach showed a vertical extension of the hypocenters enlightening the presence of sub-vertical structures, which allow the movement of the fluids towards the surface^10,11^. Within the swarms, we cannot identify any relation between number of recorded earthquakes and the maximum magnitude of the earthquakes in the swarm. On the other hand, considering the entire seismic catalog, the plot of the magnitude vs time shows that the seismicity is increasing both in terms of magnitude and rate (Fig. 1). In Fig. 1 we used the completeness magnitude Mc = 0.2 as found by Tramelli et al.^4^; this value can be considered constant since 2010 in the central part of the caldera.

### The seismicity in the context of the subsoil characteristics

We plotted the relocated earthquakes recorded between 2000 and the end of 2020 on the slices of the S-waves attenuation and Vp/Vs tomographyes obtained by Calò and Tramelly^36^ (Fig. 9 and supplementary Figure 1) and on the MT slice, ~ 5500 m long, cutting the central part of the caldera from SW to NE (Fig. 8, see^30^ for details). We choose these quantities as they can be associated to gas and/or fluid presence within the analyzed medium.

The Qs and Vp/Vs tomographic slices here represented cut the caldera from West to East with an azimuth of 110°. We distinguish a *U* shape area extending down to 2 km characterized by high attenuation (low Qs) that extends from the Pozzuoli harbor to the Solfatara/Pisciarelli area. Only the Eastern arm of this *U*, which extends below Solfatara/Pisciarelli, hosts earthquakes. The other arm is almost aseismic. These two arms have similar attenuation but different Vp/Vs: the eastern arm, where the seismicity lies, has low Vp/Vs values. This same trend is evidenced also by the Qc tomography described by Akande et al.^37^. The analyzed swarms border the bottom of this area and are placed almost at the interface between high and low Vp/Vs. The comparison of earthquake locations with the MT tomography performed by Siniscalchi et al.^30^ shows that the swarms are located at the bottom of the high resistivity vertical body extending below Solfatara/Pisciarelli (Fig. 8B). The remaining seismicity is mainly located along this high resistivity volume.

## Discussion and conclusions

The focal mechanism, which are consistent with normal, high-angle or nearly vertical faults, obtained for all the analysed earthquakes well fit with the uplift of the central part of the caldera in the analysed period.

In regions particularly enriched in water^5^, molten rocks^33^ or both of them^22^ we can assume that the faults activation is induced by the reduction of the normal stress caused by hot hydrothermal uprising fluids, as modelled by Akande et al.^37^. As a consequence, swarms and earthquakes locations can be uses to monitor fluid movements. Until 2014, a correlation between the main swarms and peaks in the geochemical signal (CO_2_/CH_4_ recorded within Solfatara crater) has indeed been reported by Chiodini et al.^38^, finding a delay of ~ 200 days.

Within fluid saturated media, as we expect for the hydrothermal system of Campi Flegrei, the temperature variations affect mostly the velocity of the P waves, modifying the fluid compressibility^26^. As a consequence, low Vp/Vs typically characterizes gas bearing rocks whereas high Vp/Vs characterizes liquid-bearing formation with low fluid compressibility^26^. In the area where the seismicity concentrates below Solfatara/Pisciarelli, Calò and Tramelli^36^ found low Vp/Vs and high attenuation (confirmed also by De Siena et al.^39^) that can be interpreted as a vertically elongated highly fractured rock matrix^40^ filled with vapor that feeds the large gas emission of Solfatara/Pisciarelli. Swarms with the higher magnitude earthquakes mainly occur in tiny volumes below this area where the presence of a rigid caprock acting as a barrier for fluids coming from a deeper magmatic source was evidenced by different authors (e.g.^41^,^39^,^36^). This layer is quite thin (~ 0.5 km^36^) and its bottom edge is characterized by high contrasts in the mechanical properties.

This interpretation is corroborated also by the comparison of earthquakes location with the MT profile proposed by Siniscalchi et al.^30^. The high resistivity vertical body extending below Solfatara/Pisciarelli between 0.5 and 2 km (Fig. 8B) was interpreted by the authors as the main upraising pathway of magmatic gases coming from the feeding reservoir located below the caprock. The earthquakes belonging to the analyzed swarms are located within or at the border of this rigid structure.

During the bradiseismic crises of 1982–84 the seismicity was spread over the caldera inner portion with two wide preferential volumes: one below Solfatara and the other extending offshore and oriented NW–SE^13^^,^^15^ To the contrary, the seismicity is currently shallower and mainly concentrated in a vertically elongated volume below the Solfatara/Pisciarelli area, even if the location of the 2012 swarm indicates that the current long-term unrest involves the entire system of the Campi Flegrei caldera. The 2012 swarm contains the deepest events and is located where the caprock should flex down^36^.

In volcanic environments, where the activation of pre-existing seismogenic sources may be the first consequence of renewed volcanic activity, it is critical to monitor the seismicity behaviour in terms of location and stress conditions. Geophysical studies performed on the 1982–84 bradiseismic crisis (e.g.^15^^,^^9^) evidenced a correlation between seismicity and variations of the pressure of the hydrothermal system. Anyway, hydrothermal instability alone cannot explain 1982–84 inflation because the densities of the estimated intrusions are greater than aqueous fluids (^20^ and references therein). A sill-like expansion source (at ~ 4 km depth) seems to be the main cause of 1970–2013 ground deformation episodes whereas the presence of a shallow hydrothermal system justifies the post 1984 mini-uplift episodes (^20^ and references therein). However, the mechanism feeding this hydrothermal reservoir is still under debate (e.g.^3,38,41^). The seismicity recorded from 2000 to 2021 is mainly located within the hydrothermal system that extends below Solfatara/Pisciarelli^25,30,36,39^, except for the main swarms that are located below this volume where the presence of a rigid caprock is regulating the injection of gases in the system above^10^. The injection of deep magmatic fluid seems to regulate the occurrence of main swarms fracturing the caprock that constitute a barrier for the magmatic fluids^38^ coming from the deep source, which feeds the shallow hydrothermal system^22^.

## Methods

Analysing the seismicity recorded since 2000 in Campi Flegrei, Chiodini et al.^25^ distinguished two populations corresponding to events occurring during swarms and the background seismicity. With the same catalogue, Tramelli et al.^4^ calculated the clustering degree that is always > 1 concluding that the seismicity occurs with swarms that are becoming denser in time. To evidence the swarms of Campi Flegrei, Bellucci Sessa et al.^42^ used the operative definition inferred by the OV seismologists, which is used locally for the surveillance activities in agreement with the Italian Department of Civil Defence. Since there is not a generally accepted quantitative definition of an earthquake swarms, except for the qualitative definition of seismic sequences with earthquakes of similar magnitude occurring close in space and time^43^, we used a method based on earthquakes rate variation^44^ to identify the main swarms. We analysed the earthquake catalogue of Campi Flegrei from 2000 to the end of 2020 using Mc = 0.2^4^. In Fig. 1C we plot the function *f(t)* defined as the difference between the number of recorded earthquakes at a certain time, *N(t)*, and the expected number, which is calculated as the rate of earthquakes (R) times the time elapsed since the beginning of the analysed catalogue:

$$f\left( t \right) = N\left( t \right) - R*t$$

The rate is estimated as the total number of earthquakes occurred in a time window divided by the window length.

As the rate of occurrence of earthquakes in Campi Flegrei increased with time since 2000^4^ we divided the catalogue into three time-series where the rate has been considered constant: [01/01/2000–13/09/2017]; [13/09/2017–07/07/2020] and [07/07/2020–31/12/2020]. These functions (black curves Fig. 1C) have been compared with the expected trends (dashed lines Fig. 1C) and the cumulative number of earthquakes (grey curve Fig. 1C). We identified 9 possible swarms and we selected 6 of them that were most significant in terms of number of events, magnitude and moment of occurrence with respect to the volcano dynamic; therefore, they were suitable for analysis. The three discarded swarms are: the swarm of October 2006 (dashed vertical line Fig. 1C), a mainly LP swarm accompanied by low magnitude earthquakes^13^^,^^14^; the September 9, 2014 swarm (first arrow Fig. 1C) composed by 9 recorded earthquakes with M_max_ = 1.0 located between Solfatara and Pozzuoli harbour and the October 12, 2018 swarm (second arrow Fig. 1C) composed by 13 recorded earthquakes with one earthquake of M = 2.0 and the others with magnitude lower than 1.1. We analysed the 6 swarms (black vertical lines in Fig. 1, summary in Table 1) focusing on their locations and focal mechanisms with respect to the seismic^36^ and MT^30^ tomographies and to the faults and fractures system^29^ to investigate the evolution of the long-term unrest which is currently taking place in the caldera.

For each swarm we relocated the earthquakes using the NLLoc program^45^ with a 3D velocity model^27^ to define the hypocentral position and for the highest magnitude earthquakes we resolved the focal mechanism using the FPFit program^46^. We reported the focal mechanisms with their misfits in the supplementary. Finally, we relocated the earthquakes using the HypoDD software^47^ which allows to define an improved position of hypocenters with respect to the others in the swarms. This allows to image possible structures involved in the swarm.

## Supplementary Information

Supplementary Information.

## Data Availability

Seismic waveforms, locations and magnitudes of Campi Flegrei earthquakes since 2016 are available at https://terremoti.ov.ingv.it/gossip/flegrei. Previous catalogue, without waveforms, is available at http://sismolab.ov.ingv.it/sismo/index.php?PAGE=SISMO/last&area=Flegrei. Waveforms prior to 2015 are currently available upon request at direzione.ov@ingv.it.
