# Supplementary material for: The seismicity of Campi Flegrei in the contest of an evolving long term unrest
Source: Sci Rep. 2022 Feb 21;12:2900. doi: 10.1038/s41598-022-06928-8 (PMC8861174; doi:10.1038/s41598-022-06928-8)
Supplement: Supplementary file 1 — Supplementary Information. [file 41598_2022_6928_MOESM1_ESM.docx]

**Supplementary materials of**

**The seismicity of Campi Flegrei in the contest of an evolving long term unrest**

Anna Tramelli^1*^, Flora Giudicepietro^1^, Patrizia Ricciolino^1^ and Giovanni Chiodini^2^


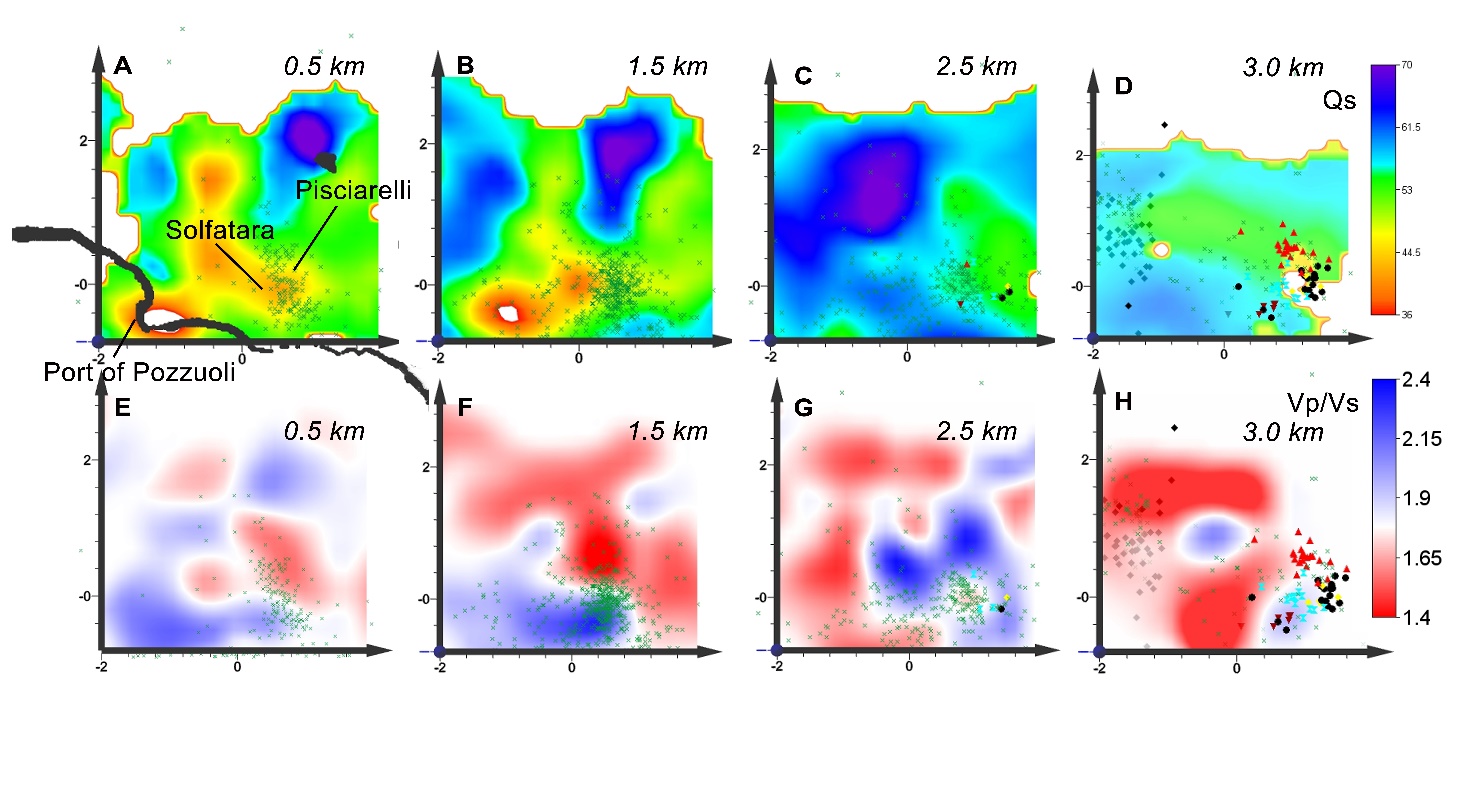


Supplementary figure 1 Horizontal sections of the Qs (top) and Vp/Vs (bottom) tomographyes of Calò and Tramelli (2019) with earthquakes and swarms. To indicate the different swarms, we used different symbols:2000-yellow diamonds; 2012-black diamonds; 2015 – bordeaux reversed triangles; 2018 – black filled circles; 2019 – red filled triangles; 2020 – hourglasses. Green crosses indicate the relocated earthquakes of the seismic catalogue between 2000 and 2020. The earthquakes plotted in each section are cut between the depth of the slice (reported at the top right of each section) and this depth + 0.5 km. The last slices (D and H) are partially transparent to allow to see also the earthquakes that occur below. Images were built with the software Voxler 3 (http://www.goldensoftware.com/products/voxler).

Supplementary table 1: Focal mechanisms of the earthquakes within the main swarms. Date is UTC in the form YYYYMMDD HHMM; latitude and longitude are degree and minutes; depth in km and strike, dip and rake and their misfits are in degree. The Δ represent the ranges of perturbations to the final solution.

| Date | Latitude | Longitude | Depth | Strike | Dip | Rake | Δstrike | Δdip | Δrake |
| --- | --- | --- | --- | --- | --- | --- | --- | --- | --- |
| 20000822 1558 | 40 49.64 | 14 8.92 | 2.64 | 30 | 60 | -80 | 18 | 8 | 20 |
| 20120907 0734 | 40 49.88 | 14 6.89 | 3.10 | 50 | 20 | -120 | 0 | 0 | 0 |
| 20120907 0825 | 40 50.01 | 14 6.92 | 3.20 | 245 | 60 | -50 | 8 | 0 | 30 |
| 20120907 0847 | 40 50.06 | 14 6.98 | 2.90 | 350 | 45 | -150 | 15 | 15 | 10 |
| 20120907 0803 | 40 50.12 | 14 7.17 | 2.91 | 0 | 40 | -140 | 10 | 13 | 5 |
| 20151007 0726 | 40 49.48 | 14 9.33 | 1.47 | 260 | 80 | -90 | 8 | 3 | 20 |
| 20151007 0731 | 40 49.88 | 14 9.09 | 2.70 | 15 | 35 | -140 | 8 | 8 | 0 |
| 20151007 0737 | 40 49.83 | 14 8.83 | 2.65 | 350 | 45 | -150 | 10 | 10 | 10 |
| 20151007 0745 | 40 49.79 | 14 9.05 | 2.55 | 30 | 55 | -150 | 18 | 8 | 5 |
| 20151007 0754 | 40 49.78 | 14 8.87 | 2.88 | 355 | 40 | -150 | 15 | 10 | 5 |
| 20151007 0802 | 40 49.73 | 14 9.19 | 2.49 | 70 | 15 | -100 | 30 | 13 | 20 |
| 20151007 0813 | 40 49.76 | 14 8.99 | 3.10 | 345 | 45 | -160 | 8 | 15 | 5 |
| 20151007 0910 | 40 49.58 | 14 8.77 | 2.64 | 5 | 55 | -160 | 20 | 15 | 5 |
| 20151007 0916 | 40 49.66 | 14 7.91 | 3.89 | 10 | 45 | -150 | 18 | 10 | 20 |
| 20180312 1337 | 40 49.94 | 14 9.28 | 2.62 | 110 | 35 | -70 | 40 | 8 | 55 |
| 20180312 1340 | 40 50.04 | 14 9.00 | 3.19 | 295 | 60 | -90 | 25 | 5 | 65 |
| 20180312 1341 | 40 49.88 | 14 8.86 | 2.44 | 255 | 60 | -60 | 3 | 3 | 0 |
| 20180312 1350 | 40 49.81 | 14 8.80 | 2.80 | 10 | 50 | -150 | 10 | 8 | 5 |
| 20180312 1358 | 40 49.98 | 14 8.85 | 2.44 | 250 | 55 | -60 | 3 | 3 | 0 |
| 20180312 1409 | 40 49.72 | 14 8.68 | 2.89 | 355 | 55 | -150 | 3 | 10 | 5 |
| 20180312 1458 | 40 49.90 | 14 8.94 | 2.70 | 15 | 45 | -140 | 10 | 8 | 10 |
| 20180312 1512 | 40 50.01 | 14 8.72 | 2.12 | 30 | 50 | -100 | 100 | 20 | 30 |
| 20191206 0004 | 40 49.74 | 14 8.81 | 2.43 | 105 | 20 | -60 | 10 | 5 | 15 |
| 20191206 0017 | 40 50.01 | 14 8.52 | 2.63 | 25 | 40 | -120 | 8 | 3 | 15 |
| 20191206 0020 | 40 49.68 | 14 8.67 | 2.41 | 50 | 25 | -130 | 40 | 10 | 40 |
| 20200426 0216 | 40 49.57 | 14 8.63 | 2.71 | 225 | 55 | -110 | 10 | 3 | 5 |
| 20200426 0241 | 40 49.56 | 14 8.76 | 2.51 | 5 | 20 | -140 | 5 | 8 | 5 |
| 20200426 0259 | 40 49.92 | 14 8.71 | 2.56 | 20 | 50 | -140 | 8 | 13 | 10 |
| 20200426 0332 | 40 49.39 | 14 8.25 | 3.76 | 60 | 60 | -30 | 33 | 8 | 20 |
| 20200426 0432 | 40 49.64 | 14 8.70 | 2.08 | 35 | 55 | -120 | 10 | 13 | 10 |
